# Tuberous Sclerosis Complex 1 Deficiency in Macrophages Promotes Unclassical Inflammatory Response to Lipopolysaccharide *In Vitro* and Dextran Sodium Sulfate-Induced Colitis in Mice

**DOI:** 10.14336/AD.2022.0408

**Published:** 2022-12-01

**Authors:** Huawen Xu, Yang Zhao, Qingjie Zhao, Mingpu Shi, Zhaoqi Zhang, Wenjun Ding, Yong Zhao

**Affiliations:** ^1^State Key Laboratory of Membrane Biology, Institute of Zoology, Chinese Academy of Sciences, Beijing, China.; ^2^Cunji Medical College, University of Chinese Academy of Sciences, Beijing, China.; ^3^Laboratory of Environment and Health, College of Life Sciences, University of Chinese Academy of Sciences, Beijing, China.; ^4^Institute for Stem Cell and Regeneration, Chinese Academy of Sciences, Beijing, China.

**Keywords:** Tuberous sclerosis complex 1 (TSC1), macrophage polarization, mammalian target of rapamycin (mtor), colitis, inflammation, lipopolysaccharide

## Abstract

Human tuberous sclerosis (TSC) is mainly caused by genetic mutations of tuberous TSC1or TSC2. Recent studies found that TSC1 deficiency promoted classical M1 macrophage polarization. However, whether TSC1 regulates other inflammatory cytokine expression in lipopolysaccharidem (LPS)-stimulated macrophages is unknown. Herein, we studied the cytokine expression profile of wild-type (WT) and TSC1-deleted macrophages after LPS stimulation in vitro and the pathogenesis of dextran sodium sulfate (DSS)-induced colitis in mice with myeloid-specific TSC1 deletion (TSC1cKO mice). We found that TSC1-deficient macrophages exhibited the enhanced secretion of interleukin-17A (IL-17A), IL-17F, and interferon-gamma (IFN-γ) in response to LPS stimulation in vitro. This is in contrast to LPS-stimulated WT macrophages, which usually do not. Importantly, TSC1cKO mice exhibited exacerbated DSS-induced acute colitis with severer symptoms. MTOR deletion or rapamycin treatment significantly reversed the enhanced expressions of IL-17A, IL-17F, and IFN-γ in LPS-stimulated TSC1-deficient macrophages in vitro and rescued the enhanced DSS-induced colitis in TSC1cKO mice, indicating that TSC1 deficiency increased these cytokine productions in an mTOR-dependent manner. RNA-sequencing and molecular studies indicated that TSC1 deficiency enhanced the aerobic glycolysis process and the activities of mTOR-STAT3-RORγT pathway in LPS-stimulated macrophages. Inhibition of aerobic glycolysis, STAT3, or RORγT reversed IL-17 and IFN-γ expression in LPS-treated TSC1-deficient macrophages. Thus, TSC1 is essential for macrophages to shut down IL-17A, IL-17F, and IFN-γ expression during LPS stimulation by suppressing the aerobic glycolysis process and mTOR-STAT3, RORγT, and T-bet pathways. The present study uncovered the key role of TSC1 in shutting down IL-17A, IL-17F, and IFN-γ expressions in LPS-treated macrophages.

The genetic mutation of tuberous sclerosis complex 1 (TSC1) or tuberous sclerosis complex 2 (TSC2) genes causes tuberous sclerosis (also called tuberous sclerosis complex, or TSC) disorder in humans, presenting with multi-organ hamartomas, renal cysts, cortical tubers, hypomelanotic macules, facial angiofibroma, progressive splenic hamartomas, subependymal giant cell astrocytoma, and drug-resistance[1-5]. TSC1 and TSC2 form a functionally heterodimeric complex to regulate cell growth, protein synthesis, cell proliferation and apoptosis, metabolism, and energy homeostasis via inhibition of mammalian target of rapamycin complex 1 (mTORC1) [6-8]. The loss of TSC1 or TSC2 leads to the constitutive hyperactivity of mTORC1 [9, 10]. mTOR inhibitors are currently used to prevent epileptogenesis in TSC patients [11]. Recently, mTOR hyperactivity was found in both colon biopsies of Crohn’s disease patients and dextran sulfate sodium (DSS)-treated mice [12]. Furthermore, it has been implicated that epithelial mTOR hyperactivation was a vicious circuit in the pathogenesis of inflammatory bowel diseases (IBD) and cancer [13]. Thus, TSC1-mTOR pathway may be involved in colitis. However, the underlying molecular mechanism and cellular components involved in TSC1-mTOR-dependent regulation of colitis remain largely elusive.

Macrophages are important components of the immune system, responsible for host defense, pathogen limitation, apoptotic cell clearing, and tissue repair [14, 15]. The classical activated macrophages (pro-inflammatory M1) mediate the pro-inflammatory response and promote the T helper type 1 (Th1)-polarized immune response against bacterial, protozoan, and viral infections by producing copious inflammatory mediators, such as tumor necrosis factor alpha (TNF-α), interleukin 12 (IL-12), and inducible nitric oxide synthase (iNOS). In contrast, the alternative activation state of macrophages (anti-inflammatory M2) is characterized by induction of a distinct set of surface receptors and effector molecules, displaying an anti-inflammatory capacity to induce type II immune responses, inflammation resolution, tissue remodeling, and wound healing. Although the M1-M2 spectrum significantly contributes to the maintenance of host defense and homeostasis, new activation phenotypes and functions of macrophages, including cytokines, growth factors, and metabolites, have been recently recognized *in vivo* and *in vitro* [16, 17]. A unique macrophage subpopulation, M (IL-23), could be induced by IL-23 and expressed a distinctive cytokine expression profile in contrast to M1 and M2 macrophages [17]. The M (IL-23) macrophage subpopulation is characterized by the production of T helper type 17 (Th17) cytokines, such as IL-17A and IL-17F, and is involved in the pathogenesis in an imiquimod-induced psoriasis mouse model [17]. Regulatory macrophages is to modulate inflammatory responses by producing anti-inflammatory mediators, and thereby limiting tissue damage [18]. Thus, the functional heterogeneity of macrophages is far beyond M1/M2 polarization. However, it is less known how molecular signal pathways master the unclassical macrophage polarization. Studies on unclassical macrophage polarization may offer novel approaches to prevent and treat the relevant inflammatory diseases.

Recent studies have highlighted the importance of the TSC-mTOR signaling pathway in the regulation of macrophage activation, contributing to various inflammatory diseases, including colitis and sepsis [3, 19, 20]. TSC1 is crucial to regulating macrophage survival, migration, and phagocytosis by mTORC1 [8]. Moreover, the TSC1 and TSC2 complex directly suppresses the Ras GTPase pathway to inhibit M1 response, and its essential role in M2 polarization is mainly mediated by inhibiting the mTOR pathway [21, 22]. However, in addition to the involvement of TSC1 in M1/M2 polarization, whether TSC1 regulates other type of macrophage polarization or other cytokine expression in LPS-stimulated macrophages is unknown. In this study, we found that TSC1-deficient but not wild-type (WT) macrophages produce large amounts of IL-17A, IL-17F, and IFN-γ after LPS stimulation *in vitro*. Molecular and biochemical mechanism studies suggested that the enhanced expression levels of IL-17A, IL-17F, and IFN-γ in TSC1-deleted macrophages are mTOR-glycolysis-dependent. Mice with a myeloid-specific deletion of TSC1 gene (TSC1cKO) displayed severe dextran sodium sulfate (DSS)-induced colitis accompanied by high levels of IL-17A, IL-17F, and IFN-γ. Our present study uncovered the essential role of TSC1-mTOR in turnoff of Th17 cytokine expression in macrophages and ameliorating acute colonic inflammation via metabolism-dependent pathways.

## MATERIALS AND METHODS

### Reagents

The monoclonal antibodies (mAbs) for flow cytometry analysis used in this study are listed: anti-mCD11b-BV510 (Cat#: 101245; BioLegend), anti-mCD45-BUV395 (Cat#: 564279; BD), anti-mCD45-PE-Cy5 (Cat#: 103132; BioLegend), anti-mLy6G-FITC (Cat#: 127606; BioLegend),anti-mF4/80-PE-Cy5 (Cat#: 15-4801-82; Invitrogen), anti-mF4/80-PE (Cat#: 123110; BioLegend), anti-mIL-17A-PE (Cat#: 506904; BioLegend), anti-mIL-17A-BV421 (Cat#: 506925; BioLegend), anti-mIFN-γ-PE(Cat#: 505808; BioLegend), anti-mIFN-γ-FITC (Cat#: 505806; BioLegend), anti-m CD16/CD32 (2.4G2, Cat#: 553142; BD Pharmingen™). There are antibodies used for western blot assay: anti-β-Actin (AC-15; Cat#: ab6276; abcam), phospho-Stat3(Tyr705) (Cat#: 9131S; Cell signaling), phospho-Stat3 (Ser727) (Cat#: 9136S; Cell signaling), ROR gamma (t) (Cat#: 14-6988-82; eBioscience™), phospho-S6 (Ser235/236) (Cat#: 2211S; Cell signaling), phospho-eIF4E (Ser209) (Cat#: 9741S; Cell signaling), eIF4E (C46H6) (Cat#: 2067S; Cell signaling), anti-rabbit secondary antibodies (Cat#: 111-035-003; Jackson ImmunoResearch Laboratories), prestained protein ladder (Cat#: 26616; PageRuler™). Other major reagents used in this study: Recombinant murine M-CSF (Cat#: 315-02-100; PeproTec), LPS (Cat#: L2630; Sigma). All reagents were used following the manufacturers’ instructions.

### Animals

**Figure 1.**
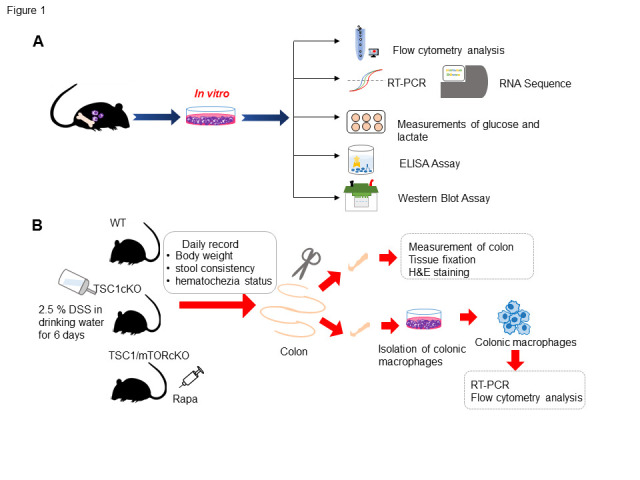
Graphic outline of *in vivo* and *in vitro* experiments. A flowchart of the in vitro experiments (A). Schematic representation of the protocol of DSS induced colitis in mice (B). WT, wild type; TSC1cKO, myeloid-specific TSC1 deletion; mTOR, mammalian target of rapamycin; RT-PCR, Real time-polymerase chain reaction; DSS, dextran sodium sulfate; Rapa, rapamycin; H&E, hematoxylin and eosin; ELISA, enzyme-linked immunosorbent assay.

Mice with TSC1 deletion in their myeloid cells (LysM-CreTsc1^loxp/loxp^; TSC1cKO) were generated by crossbreeding TSC1^loxp/loxp^ mice with LysM-Cre mice expressing Cre recombinase under the control of Lysozyme promoter (LysM-Cre) [21, 23]. The WT littermates served as the control. TSC1/mTORcKO mice, with TSC1 and mTOR double deletion (LysM-CreTSC1^loxp/loxp/^mTOR^loxp/loxp^; TSC1/mTORcKO) in myeloid cells, were obtained from crossbreeding TSC1^loxp/loxp^ and mTOR^loxp/loxp^ mice. The 4-5-week-old C57BL/6J mice and 6-8-week-old female CD45.1 C57BL/6J mice were purchased from Beijing University Experimental Animal Center (Beijing, China). The TSC1^loxp/loxp^ mice were gifts from Dr. Hongbin Zhang (Institute of Basic Medical Sciences and School of Basic Medicine, Peking Union Medical College, and Chinese Academy of Medical Sciences, Beijing, China). The LysM-Cre mice were kindly offered by Dr. Lianfeng Zhang (Key Laboratory of Human Diseases Comparative Medicine, Ministry of Health, Institute of Laboratory Animal Science, CAMS & PUMC). All mice were bred and maintained in the specific-pathogen-free animal facility of the Animal Experiment Centre of the Institute of Zoology in Beijing, China. All experiments were ethically performed according to the Institute of Zoology’s Institutional Guidelines for the Care and Use of Laboratory Animals. The flowchart and procedures of the in vitro and in vivo experiments were shown in Fig. 1.

### Cell culture of macrophages

The euthanasia method for mice used in this study is carbon dioxide asphyxiation which mice were placed in a new cage and then euthanized by the air of 100% carbon dioxide inhalation [24]. The bone marrow (BM) cells were collected and cultured in high-glucose Dulbecco’s Modified Eagle Medium (DMEM) supplemented with 5% fetal bovine serum (FBS) and 0.2% mouse macrophage colony-stimulating factor (M-CSF) for macrophage differentiation for 14 days at 37 °C with 5% CO_2_ to induce bone marrow-derived macrophages (BMDM) [20, 21, 25]. The peritoneal cavity cells were freshly obtained from the peritoneal cavity of mice with phosphate-buffered saline (PBS). The peritoneal macrophages (PEMs) were adherent through being cultured in plates at 37 °C with 5% CO_2_ for 2 h [26]. The purified peritoneal macrophages were obtained via discarding the supernatant. After 2 warm PBS washes, the peritoneal macrophages were incubated at 37 °C with 5% CO_2_ with stimulations for an indicated amount of time.

**Table 1:** Primers used for RT-PCR analysis.

Genes	Primer sequence (5’ — 3’)
TNF-α-Forward	GAGTGACAAGCCTGTAGCC
TNF-α-Reverse	CTCCTGGTATGAGATAGCAAA
IL-12-Forward	CACGGCAGCAGAATAAATA
IL-12-Reverse	CTTGAGGGAGAAGTAGGAATG
IL-17A-Forward	CTCAGACTACCTCAACCGTTCC
Il-17A-Reverse	ATGTGGTGGTCCAGCTTTCC
IL-17F-Forward	CATACCCAGGAAGACATACTTAGAAG
IL-17F-Reverse	AGTCCCAACATCAACAGTAGC
IFN-γ-Forward	GAACTGGCAAAAGGATGGTGA
IFN-γ-Reverse	TGTGGGTTGTTGACCTCAAAC
IL-23-Forward	CTGAGAAGCAGGGAACAAGATG
IL-23-Reverse	GAAGATGTCAGAGTCAAGCAGGTG
T-bet-Forward	AGCAAGGACGGCGAATGGT
T-bet-Reverse	GGGTGGACATATAAGCGGTTC
Ldhb-Forward	TGCGTCCGTTGCAGATGAT
Ldhb-Reverse	TTTCGGAGTCTGGAGGAACAA
Eno1-Forward	TGCGTCCACTGGCATCTAC
Eno1-Reverse	CAGAGCAGGCGCAATAGTTTTA
Gpi1-Forward	CTCAAGCTGCGCGAACTTTTT
Gpi1-Reverse	GGTTCTTGGAGTAGTCCACCAG
Pfkm-Forward	GCGACTTGCTGAATGATCTCC
Pfkm-Reverse	CATTGTCGATTGAGCCAACCA
Aldoc-Forward	AGAAGGAGTTGTCGGATATTGCT
Aldoc-Reverse	TTCTCCACCCCAATTTGGCTC
HK1-Forward	CGGAATGGGGAGCCTTTGG
HK1-Reverse	GCCTTCCTTATCCGTTTCAATGG
HK3-Forward	TGCTGCCCACATACGTGAG
HK3-Reverse	GCCTGTCAGTGTTACCCACAA
HPRT-Forward	AGTACAGCCCCAAAATGGTTAAG
HPRT-Reverse	CTTAGGCTTTGTATTTGGCTTTTC

### DSS-induced colitis mouse model

Colitis was induced in WT and TSC1cKO mice via 2.5% DSS (M.W. = 36,000-50,000,Ref = 160110) in drinking water for 6 days [27, 28]. Each group consisted of 5-6 mice with a control group who were given drinking water without DSS. The mice were monitored daily to observe their condition, including physical appearance, diarrhea, and body weight change [29]. The disease activity index (DAI) score, composed of body weight change, diarrhea, and hematochezia (or characteristics of the stool and occult blood in the stool), was recorded every day after the administration of DSS (Table 1). DAI = [(score of body weight loss) + (score of stool consistency) + (score of hematochezia status)]/3. The mice were killed on the sixth day after DSS or drinking water administration. Their colon was removed, measured, and assessed for signs of damage.

### Isolation of colonic macrophages

Colonic macrophages were collected from the mice colons by enzymatic digestion based on the established method [30]. The colon was cut into pieces and washed with DMEM, then the colon fragments were resuspended in 2 ml solution of 1 mg/ml collagenase/dispase (Sigma-Aldrich, USA) containing 20 U/ml DNAse I (Sigma-Aldrich, USA), and incubated at 37 °C for 30 min. PBS with 1% FBS and 5 mM ethylenediaminetetraacetic acid (EDTA) was used to neutralize digestion. Cells were centrifuged at 1,700 rpm for 5 min at 4 °C, resuspended in DMEM, and then filtrated to remove clumps. The isolated cells were seeded in 24-well plates and cultured for 2 h in the incubator supplied with 5% CO_2_ to obtain adherent colonic macrophages.

### Hematoxylin and eosin (H&E) staining of colon

H&E staining was performed on the basis of the protocol from previous studies [31, 32]. In brief, divided colon sections (1 cm) from TSC1cKO mice and littermate WT mice were thoroughly rinsed with PBS buffer and then fixed using 4% formalin at room temperature. Then, the colon sections were embedded in paraffin and sectioned into 4 µm thickness slices. The colon tissue slices were subsequently stained by H&E and examined via Aperio VERSA (Leica) [33].

### Rapa treatment in vivo

The 4-5-week-old TSC1cKO and WT mice were injected intraperitoneally with Rapa (50 μg/kg body weight) or carboxymethyl cellulose sodium (CMC) for 3 days.

### Flow cytometry analysis

To analyze the production of IL-17A and IFN-γ by CD11b^+^F4/80^+^ macrophages, PEMs treated *in vitro* were plated in 24-well plates at a density of 1 X 10^6^/ml and incubated for 6 h at 37 °C with 5% CO_2_. Single-cell suspensions were prepared and then washed once using cold PBS blocked with anti-CD32/CD16 to avoid nonspecific binding [34]. They were then stained with anti-mouse antibodies of cell surface markers, including CD45, CD11b, F4/80 and Ly6G, for 30 min at 4 °C. For intracellular IL-17A and IFN-γ staining, macrophages were co-cultured with LPS (100 ng/ml) and GolgiPlug (BD Pharmingen) for 6 h. BD Cytofix/Cytoperm and BD Perm/Wash buffer set were used according to the manufacturer’s instructions (BD Pharmingen, USA). Data acquisition was performed with BD LSRFortessa™ cell analyzer (BD Biosciences, USA), and then data were analyzed via FlowJo software (Tree Star, USA).

### ELISA assay of IL-17A

Cell culture supernatants were examined for IL-17A with Mouse IL-17A ELISA Kits (BioLegend, USA) according to the manufacturer’s instructions [35]. The 96-well plate coated with diluted capture antibody solution was prepared in advance on day 1. On day 2, the plate was blocked with 1X assay diluent A at room temperature for 1 h with shaking (500 rpm) in the dark after washing 4 times with wash buffer (1X). After the 4 washes, prepared standards and samples were added to the plate, sealed, and incubated at room temperature for 2 h while shaking. Then, the detection antibody solution was added for 1 h, followed by Avidin-HRP solution for 30 min. The substrate solution F was used, and the reaction was stopped by stop solution for 15 min in the dark. Optical absorbance was determined at 450 nm using a spectrophotometer (Agilent, USA).

### RNA extraction and quantitative PCR analysis

Total mRNA was extracted using Trizol reagent (Invitrogen, CA) or a MicroElute Total RNA kit (OMEGA bio-tek, USA) according to the manufacturers’ instructions [36]. RNA quality and quantity were assessed using a NanoDrop 2000 (Thermo Fisher Scientific). Freshly extracted mRNA was immediately reverse-transcribed to cDNA with M-MLV superscript reverse transcriptase according to the manufacturer’s instructions. Real-time PCR was performed using SYBR Premix Ex Taq on a real-time fluorescence quantitative PCR instrument (Roche, Basel, Switzerland). All reagents were used according to the manufacturer’s instructions. The mRNA expression levels of each gene were normalized to the expression level of the housekeeping gene to calculate results. The primers used in the present study are listed in Table 1. Data were analyzed by 2^-ΔΔCt^ method.

### RNA sequencing and functional analysis

We prepared the total mRNA as described above and followed the reported method for data processing [37, 38]. Processed reads were aligned to mouse genome (mm10) via hierarchical indexing for spliced alignment of transcripts (HISAT2) [39]. String Tie was used to assemble and quantify the transcripts in each sample to obtain the number of exon, transcription initiation/stop site, count, and transcripts per kilobase of exon model per million mapped reads (TPM) values. The identification of differential expression genes (DEGs) was performed by using the DEseq2 [40] R-packages with count values. The threshold is under the condition of adjusted *P*-value < 0.05 and |Foldchange| > 2. We applied Kyoto Encyclopedia of Genes and Genomes (KEGG) PATHWAY [41] (available online: www.genome.jp/kegg) and KEGG Orthology-Based Annotation System (KOBAS) 3.0 [42] (available online: http://kobas.cbi.pku.edu.cn/) to perform pathway analysis. In addition, gene set enrichment analysis (GSEA) was carried out using the REACTOME database [43]. The analyses were selected with p < 0.05 as the cutoff criterion. A transcriptional interaction network was created using STRING [44] (available online: https://string-db.org/) and visualized by Cytoscope [45].

### Measurements of glucose and lactate

Glucose and lactate levels were immediately determined after macrophages (2 x 10^6^ cells/well) seeded in a 24-well plate were incubated for 2 days *in vitro*. Supernatants of cell culture medium were collected, and then the glucose and lactate levels were measured using the 1450 MicroBeta JET (Perkin Elmer) [46]. Glucose consumption and lactate production were normalized to cell numbers. The experiments were performed in triplicate and independently repeated 3 times.

### Western blot

Macrophages cells (2×10^6^ cells/well) were cultured in DMEM medium with 5% FCS in 24-well plates. The cells were treated with indicated stimulus, LPS (100 ng/ml) or Rapamycin (100 nM) or LPS and Rapamycin, for the indicated time. After stimulation, the cells were washed twice with cold PBS and then lysed in RIPA buffer (50 mM Tris-HCl pH 7.4, 1% NP-40, 0.25% Na-deoxycholate, 150 mM NaCl, 1 mM EDTA pH 7.4) with protease and phosphatase inhibitor cocktails (Cat#: PPC1010, Sigma) for 10 mins on a rocker at 4 °C. The protein concentration was determined via BCA assay. The protein contents were separated by SDS polyacrylamide gel electrophoresis (SDS-PAGE) and then transferred onto PVDF membranes (Millipore, CA). Each polyvinylidene fluoride membrane was blocked with TBST (100 mM Tris-HCl pH 7.5, 150 mM NaCl, 0.05% Tween20) in 5% skim milk for 1 hour at room temperature, then incubated with primary antibodies overnight on a shaker at 4 °C. Subsequently, HRP-coupled secondary antibody was added, and protein samples were detected by chemiluminescence (Millipore, CA). Anti-β-actin mAb (working concentration: 1:10000) determined in previous studies was chosen as a protein loading control [17].

### Statistical analysis

Data were analyzed using GraphPad Prism 8.3.0, SPSS 21.0 and Microsoft Excel 2010 software. Data are expressed as mean ± standard deviation (SD) or mean ± standard error of the mean (SEM) as appropriate, which is representative of at least 3 independent experiments with a minimum of 3 biological replicates. Log-rank tests were used for mouse survival assays. Kolmogorov-Smirnov and F-value tests were performed to analyze normal distribution and homogeneity of variance of data in order to determine normality of data. Student’s unpaired two-tailed t-test for comparison between two groups, or one-way ANOVA analysis for multiple groups were used as paramedic methods. Mann-Whitney U test was used as the none-paramedic method. *P*<0.05 was considered statistically significant.

**Figure 2.**
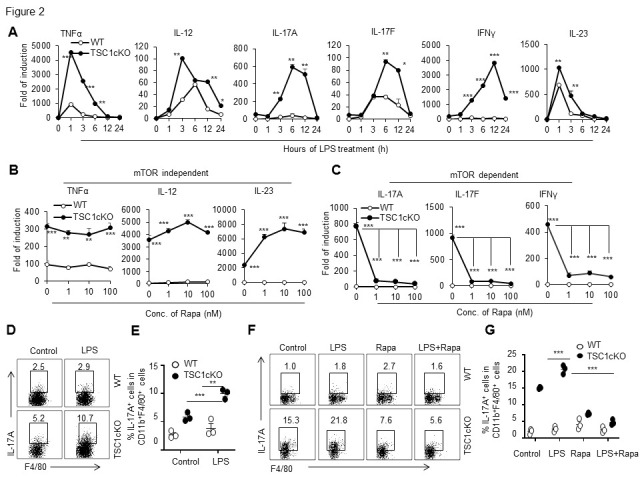
Lipopolysaccharide-stimulated tuberous sclerosis complex 1-deficient macrophages expressed interleukin-17A, interleukin-17F and interferon-gamma in mammalian target of rapamycin-dependent manner. WT and TSC1cKO peritoneal macrophages freshly isolated from the muse peritoneal cavity were stimulated with LPS (100 ng/ml) at indicated time points. Expression of TNF-α, IL-12, IL-23, IL-17A, IL-17F and IFN-γ was detected by real-time PCR (mean ± SD, n=3 biological replicates, Student’s t-test) (A). Isolated peritoneal macrophages were firstly pre-cultured with Rapa (1, 10 and 100 nM) for 90 mins and then cultured with LPS (100 ng/ml) for additional 6h. MRNA expression levels of TNF-α, IL-12, IL-23, IL-17A, IL-17F, and IFN-γ in the treated macrophages was determined by real-time PCR (mean ± SD, n=3 biological replicates, one-way ANOVA test) (B, C). Flow cytometric analysis and quantification of IL-17A expression in the CD11b^+^F4/80^+^ cell population stimulated with LPS (100 ng/ml) for 6h (mean ± SEM, n=3 biological replicates, Student’s t-test) (D, E). The PEMs were collected in the sixth day after the injection of 1 ml 0.3% (w/v double-distilled water) BBL™ thioglycolate medium brewer modified (TG) in peritoneal cavity. Rapa pre-treatment for 90 min and then LPS stimulation for 6 h *in vitro* and the production of IL-17A was detected by flow cytometry (mean ± SEM, n=3 biological replicates, one-way-ANOVA analysis) (F, G). Statistically significant results are labeled: * *p* < 0.05, ** *p* < 0.01, *** *p* < 0.001 compared with WT mice or between the indicated groups. IL, interleukin; LPS, lipopolysaccharide; mTOR, mammalian target of rapamycin; IFN-γ, interferon gamma; TNF-α, tumor necrosis factor alpha; TSC1, tuberous sclerosis complex 1.

**Figure 3.**
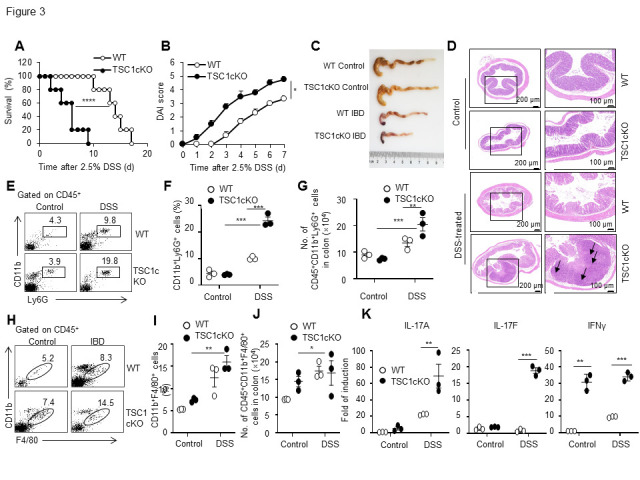
Mice with a myeloid-specific tuberous sclerosis complex 1 deletion suffered from severer dextran sodium sulfate-induced colitis than wild-type mice. Acute colitis was induced using 2.5% dextran sulfate sodium (DSS) in drinking water for 6 days. Mortality rate of the mice with colitis was recorded (n= 5 mice in each group, long-rank test) (A). DAI scores of the mice were scored daily after the treatment of DSS (mean ± SEM, n=5 mice in each group) (B). Picture of WT and TSC1cKO colons after 6 days of 2.5% DSS administration. The shorter colon length was presented in DSS-treated mice (C). Colon tissue specimens were fixed, embedded in paraffin, sectioned and stained with hematoxylin and eosin (H&E). Representative H&E staining images of colonic tissues show longitudinal sections. Infiltration of inflammatory cells, mucosal necrosis, thinner serous membrane and thicker lamina propria observed in the colon following induction of colitis with DSS treatment. The black arrows point to the pathophysiological changes (D). Flow cytometry analysis of CD11b^+^Ly6G^+^ neutrophils (mean ± SEM, n=3 biological replicates) (E-G) and CD11b^+^F4/80^+^ macrophages (mean ± SEM, n=3 biological replicates) (H-J) in WT and TSC1cKO colon. (K) The mRNA expression of IL-17A, IL-17F and IFN-γ of isolated colonic macrophages (mean ± SEM, n=3 biological replicates). Data in (B), (F-G) and (J-K) were analyzed by Students’ t-test. Data in (I) were analyzed by Mann-Whitney U test. *p <0.05, ** *p* < 0.01, *** *p* < 0.001 compared with the control or between the indicated groups. DSS, dextran sodium sulfate; TSC1, tuberous sclerosis complex 1; WT, wild-type; IL, interleukin; IFN-γ, interferon gamma.

## RESULTS

### TSC1 inhibited Th1/Th17 cytokine expression in LPS-stimulated macrophages via an mTOR-dependent fashion

Myeloid cell-specific TSC-deleted mice were generated by crossbreeding TSC1^loxp/loxp^ mice with transgenic mice that carried lysozyme (LysM) proximal promoter-mediated Cre recombinase (LysM^Cre^TSC1^loxp/loxp^; herein referred to as TSC1cKO). Then we detected the mRNA expression levels of a set of cytokines in freshly isolated wild type (WT) and TSC1cKO mouse peritoneal resident macrophages after LPS stimulation for 6 h by real-time PCR. The LPS treatment induced significantly higher tumor necrosis factor-α (TNF-α), interleukin 12p40 (IL-12p40), and interleukin 23 (IL-23) mRNA expression in freshly isolated TSC1cKO peritoneal macrophages compared with WT macrophages (Fig. 2A). Unexpectedly, LPS induced mRNA expression in Th1/Th17-type cytokines, including IL-17A, IL-17F, and IFN-γ, in TSC1-deficient macrophages in a time-dependent manner, as determined by real-time PCR (Fig. 2A). It is well known that TSC1 deficiency in macrophages had higher mTOR activity. We thus investigated the possible role of mTOR in the TSC1-mediated regulation of these cytokine expressions in LPS-stimulated peritoneal macrophages. We found that blocking mTOR with low and high doses of rapamycin (Rapa) for 90 min pretreatment failed to inhibit the enhanced TNF-α, IL-12, and IL-23 expression in TSC1-deficient macrophages (Fig. 2B). However, Rapa treatment significantly reversed the enhanced expressions of IL-17A, IL-17F, and IFN-γ in TSC1-deficient macrophages, even with low doses of Rapa (Fig. 2C). To further determine the role of TSC1 in controlling IL-17A protein expression in macrophages, we detected the IL-17A protein expression in LPS-treated TSC1cKO macrophages using flow cytometry. A significantly higher percentage of IL-17A^+^ cells in TSC-deficient macrophages was observed after LPS stimulation compared with WT macrophages (Fig. 2D-E). Furthermore, mTOR inhibition by Rapa significantly reversed the IL-17A expression in TSC1cKO macrophages, as determined by flow cytometry assays (Fig. 2F-G). These results collectively suggest that TSC1 deficiency in macrophages promotes IL-17A, IL-17F, and IFN-γ expression in response to LPS in an mTOR-dependent manner.

**Figure 4.**
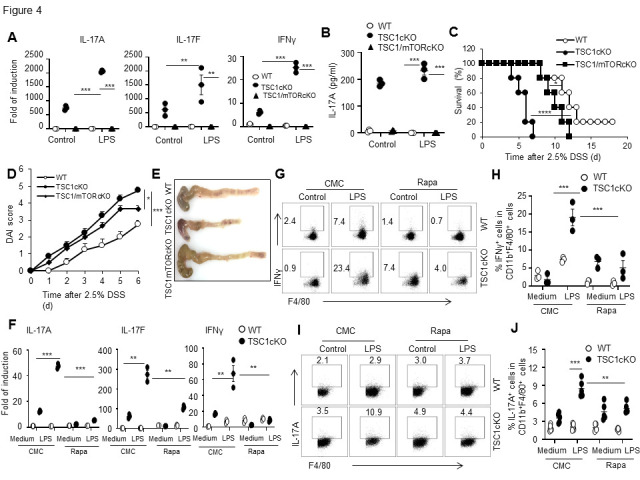
Mammalian target of rapamycin deletion or rapamycin treatment reversed the enhanced T helper type 1/T helper type 17 cell-like cytokine expression in tuberous sclerosis complex 1-deficient macrophages and the severer dextran sodium sulfate-induced colitis in mice with a myeloid-specific tuberous sclerosis complex 1 deletion. The mRNA expression of IL-17A, IL-17F and IFN-γ in the sorted CD45^+^CD11b^+^F4/80^+^ macrophages after LPS (100ng/ml, 6h) stimulation (A). The concentrations of IL-17A in the culture medium were determined by ELISA (B). After 2.5% DSS administration for six days, TSC1/mTORcKO mice had significantly higher survival percentage (C) and lower DAI scores (D) than TSC1cKO mice. The image of WT, TSC1cKO and TSC1/mTORcKO colons on day 6 (E). The mRNA expression of IL-17A, IL-17F, and IFN-γ following the treatment of Rapa. Both WT and TSC1cKO mice were injected with Rapa (50 μg/kg body weight) for three days, PEMs obtained and stimulated with LPS for 6 h (F). The IFN-γ^+^ cells were analyzed in CD11b^+^F4/80^+^ cells after Rapa pre-treatment and LPS stimulation *in vitro* (G). The percentage of IFN-γ^+^ cells in CD11b^+^F4/80^+^ cells (H). The IL-17A^+^ cells were analyzed in CD11b^+^F4/80^+^ cells after Rapa pre-treatment and LPS stimulation *in vitro* (I). The percentage of 17A^+^ cells in CD11b^+^F4/80^+^ cells (mean ± SEM, n=5 biological replicates) (J). Data in (C) from 5 mice in each group was analyzed by Long-rank test. Data in (D) from 5 mice in each group was used Students’ t-test. Data, displayed as dot plots, in (A, B, H, F) are present as mean ± SEM (n=3 biological replicates, one-way-ANOVA analysis). Statistical significance is labelled in the figure (*p < 0.05, **p <0.01, ***p < 0.001). DAI, disease activity index; DSS, dextran sodium sulfate; LPS, lipopolysaccharide; mTOR, mammalian target of rapamycin; TSC1, tuberous sclerosis complex 1; IL, interleukin; IFN-γ, interferon gamma.

**Figure 5.**
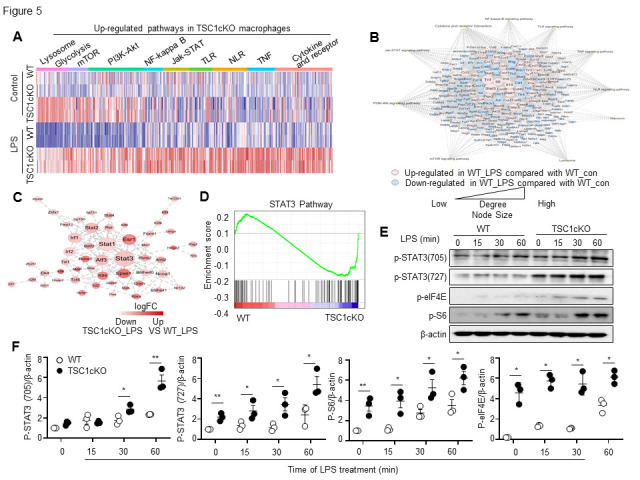
Tuberous sclerosis complex 1 deficiency enhanced signal transducer and activator of transcription 3 signal pathways in lipopolysaccharide-treated macrophages. Classification of up-regulated genes and KEGG enriched pathways after TSC1cKO under LPS stimulation (A). Network analysis summarized the changes in WT cells stimulated and unstimulated by LPS in the previous KEGG enrichment pathway (B). Network analysis of upregulated transcription factors in TSC1 knockout macrophages (C). STAT3 signaling pathway in WT and TSC1cKO macrophages was analyzed using GSEA tools (D). Isolated primary macrophages from WT and TSC1cKO mice were treated with LPS (100 ng/ml) at indicated time points. The levels of p-STAT3, p-eIF4 and p-S6 were analyzed by western blot (E). Western blot quantification analysis by ImageJ (F). Data are expressed as mean ± SEM (n=3 times experiments, Students’ t-test, *p < 0.05, **p <0.01). PI3K-AKT, phosphatidylinositol 3-kinase-protein kinase B; TLR, toll-like receptor; NLR, NOD-like Receptor; JAK, Janus kinase; TNF, tumour necrosis factor; p-eIF4E, phosphorylated-eukaryotic translation initiation factor 4E; p-S6, phosphorylated S6 ribosomal protein; KEGG, Kyoto Encyclopedia of Genes and Genomes; LPS, lipopolysaccharide; TSC1, tuberous sclerosis complex 1; STAT3, signal transducer and activator of transcription 3.

### Mice with myeloid-specific TSC1 deletion are more sensitive to DSS-induced colitis

To investigate the physiological significance of the IL-17A and IFN-γ-producing macrophages mediated by TSC1 deficiency *in vivo*, we used a DSS-induced acute colitis mouse model in which IL-17A and IFN-γ have been reported to play critical roles [20, 24, 47-49]. After feeding WT and TSC1cKO mice with water containing 2.5% DSS daily, most WT mice survived for at least 10 days, but all TSC1cKO mice died within 10 days (*P* < 0.01, Fig. 3A). Moreover, the TSC1cKO mice displayed more severe symptoms of acute colitis than the WT mice as indicated by the increased macroscopic scores (Fig. 3B), and shorter colon lengths (Fig. 3C). Pathological tests after 6 days of DSS treatment revealed severe colitis in the TSC1cKO mice, including massive infiltration of the mucosal layer and lamina propria and thickened lamina propria (Fig. 3D). In addition, more CD11b^+^Ly6G^+^ neutrophil infiltration was observed in the colons of the DSS-treated TSC1cKO mice than in those of the WT mice (Fig. 3E-G). Although the percentages and cell numbers of CD11b^+^F4/80^+^ macrophages in the colons of DSS-treated TSC1cKO mice were similar to those in DSS-treated WT mice (Fig. 3H-J), the freshly isolated colonic macrophages from DSS-treated TSC1cKO mice displayed higher levels of IL-17A, IL-17F, and IFN-γ expression, indicating the potential unclassical inflammatory polarization status of macrophages *in vivo* (Fig. 3K).

**Figure 6.**
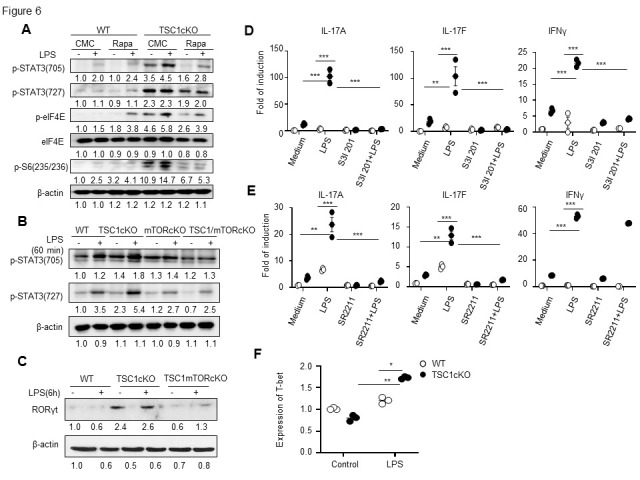
The enhanced signal transducer and activator of transcription 3- retineic-acid-receptor-related orphan nuclear receptor gamma signal pathways is involved in T helper type 1/T helper type 17 cell cytokine expression in lipopolysaccharide-treated tuberous sclerosis complex 1-deficient macrophages. WT and TSC1cKO mice (n=3 mice in each group) were firstly treated with Rapa (50 μg/kg) for three days *in vivo*, then primary macrophages were isolated to determine the change in p-STAT3, p-S6 and p-eIF4E after LPS treatment by western blot (A). Peritoneal macrophages from WT, TSC1cKO, mTORcKO and TSC1/mTORcKO were treated with LPS for 60 min *in vitro* and the p-STAT3 level was determined by western blot (B). Western blot analysis of cell lysates to detect RORγT (C). The mRNA expression of L-17A, IL-17F and IFN-γ was determined after the stimulation of S3I201 and LPS, respectively (mean ± SEM, n=3 biological replicates) (D). The mRNA expression of IL-17A, IL-17F and IFN-γ was ascertained after the pre-treatment of SR2211 (10 μM) for 0.5h and then stimulation of LPS (100ng/ml) for 6 h (mean ± SEM, n=3 biological replicates) (E). Relative mRNA level of T-bet was determined after LPS stimulation by real-time PCR (mean ± SEM, n=3 biological replicates) (F). Data in (D-F) were analyzed by one-way ANOVA analysis. Statistical significance is shown: *p < 0.05, **p <0.01, ***p < 0.001 compared with the control or between the indicated groups. STAT3, signal transducer and activator of transcription 3; TSC1, tuberous sclerosis complex 1; IL, interleukin; IFN-γ, interferon gamma LPS, lipopolysaccharides; P-eIF4E, phosphorylated-eukaryotic translation initiation factor 4E; P-S6, phosphorylated S6 ribosomal protein.

### MTOR deletion or Rapa treatment reversed the enhanced Th1/Th17-like cytokine expression in TSC1-deficient macrophages and the severer DSS-induced colitis in mice with a myeloid-specific TSC1 deletion

To study the role of mTOR in the enhanced expression of IL-17A, IL-17F, and IFN-γ in TSC1cKO macrophages and the more severe symptoms of acute colitis in TSC1cKO mice, we produced mice with mTOR deletion (LysM^Crem^TOR^loxp/loxp^ or mTORcKO) or with TSC1 and mTOR double deletion (LysM^Cre^TSC1^loxp/loxp^/mTOR^loxp/loxp^; herein referred to as TSC1/mTORcKO) in myeloid cells. Peritoneal macrophages isolated from TSC1/mTORcKO mice expressed almost undetectable levels of IL-17A, IL-17F, and IFN-γ, the same as the WT and mTOR deletion cells after LPS stimulation as determined by real-time PCR (Fig. 4A). TSC1cKO macrophages produced more IL-17A, whereas TSC1/mTORcKO macrophages produced IL-17A less than the WT macrophages after LPS stimulation, as detected in the IL-17A levels in the supernatants by ELISA assays (Fig. 4B). Importantly, mTOR deletion can reverse the more severe symptoms of acute colitis in TSC1cKO mice, as shown by significantly extended survival time (Fig. 4C), decreased macroscopic scores (Fig. 4D), and longer colon length (Fig. 4E) in TSC1/mTORcKO mice compared with TSC1cKO mice. Peritoneal macrophages isolated from WT and TSC1cKO mice pretreated with Rapa (50 μg/kg body weight) *in vivo* for 3 days showed low levels of IL-17A, IL-17F, and IFN-γ expression in mRNA levels after LPS stimulation (Fig. 4F). Flow cytometric results also showed that mTOR inhibition by rapamycin in vivo significantly decreased the enhanced percentages of IL-17A^+^ and IFN-γ^+^ cells in CD11b^+^F4/80^+^ macrophages (Fig 4G-J). Altogether, the increased mTOR activity caused by TSC1 deficiency in macrophages might contribute to the accelerated DSS-induced colitis in TSC1cKO mice and high expression of IL-17A, IL-17F, and IFN-γ in macrophages.

### Loss of TSC1 in macrophages exhibited an upregulated inflammatory gene expression profile

To characterize the molecular state associated with the different functional activities of LPS-stimulated TSC1cKO macrophages versus LPS-stimulated WT macrophages, we performed RNA-sequencing (RNA-seq) on LPS-stimulated WT and TSC1cKO macrophages. Through differential gene analysis, we found that compared with WT macrophages, TSC1 deficiency in macrophages led to the upregulation of 2,451 genes and downregulation of 1,651 genes under LPS stimulation. Among them, the upregulated genes were mainly enriched in cytokine interaction pathways (cytokine and cytokine receptor interaction and Jak-STAT signaling pathway), macrophage pattern recognition receptor-related pathways NOD-like Receptor (NLR) signaling pathway and TLR-like Receptor (TLR) signaling pathway), inflammatory signaling pathways (TNF signaling pathway and NF-kappa B signaling pathway), and metabolism-related signaling pathways (PI3K-Akt signaling pathway, mTOR signaling pathway, Glycolysis, and Lysosome) (Fig. 5A). The abovementioned genes, whose expression was upregulated in LPS-stimulated TSC1cKO macrophages, were used to construct a gene pathway network analysis (Fig. 5B). Among them, pattern recognition receptor, cytokine interaction, and inflammatory signaling pathways were upregulated after LPS stimulation, whereas metabolism-related pathways were unchanged or downregulated after LPS stimulation. Knocking out TSC1 can promote inflammatory responses by promoting the positive and negative expression of genes after LPS stimulation. In particular, the cytokines-JAK-STAT (interleukin 6 (Il6), interleukin 10 (Il10), interleukin 18 (Il18), Tnf, Stat1, Stat3) network showed the strongest interaction capacity at the center of all gene networks. Subsequently, we constructed a transcription factor network and found that signal transducer and activator of transcription 3 (STAT3) was located in an important central position in the upregulated transcription factor network in TSC1cKO macrophages (Fig. 5C). Next, gene set enrichment analysis (GSEA) also suggested that STAT3 and its related signaling pathway were significantly upregulated in TSC1cKO macrophages compared with WT macrophages (Fig. 5D). Additionally, western blot analysis showed that TSC1 deficiency promoted STAT3 activation in LPS-stimulated macrophages based on the enhanced levels of p-STAT3 (Y705 and S727, Fig. 5E-F). These results led us to speculate that the STAT3 signaling pathway may be involved in the Th1/Th17-like inflammatory response in LPS-stimulated TSC1cKO macrophages.

### TSC1 blocked Th1/Th17 cytokine expression in macrophages by decreasing mTOR-STAT3-RORγT and T-bet pathways

Rapa treatment or mTOR deletion decreased the enhanced levels of STAT3 activation caused by TSC1 deficiency in macrophages, as indicated by the p-STAT3 levels found by western blot analysis (Fig. 6A-B). Rapa treatment decreased eIF4E and S6 activation in TSC1cKO macrophages, which were both downstream of TSC1-mTORC1, as indicated by the levels of phosphorylated-eukaryotic translation initiation factor 4E (p-eIF4E) and phosphorylated S6 ribosomal protein (P-S6; Ser 235/236) detected by western blot analysis (Fig. 6A). It has been reported that RORγT is a critical transcription factor for Th17 cells and can be regulated by STAT3. Thus, we observed the protein expression of RORγT in WT and TSC1cKO macrophages. Western blot analysis showed that the RORγT protein expression was upregulated in TSC1cKO macrophages compared with those in WT macrophages, which could be inhibited by mTOR deletion (Fig. 6C). Inhibition of STAT3 activation by the STAT3-specific inhibitor S31-201 significantly decreased IL-17A, IL-17F, and IFN-γ mRNA expression in TSC1cKO macrophages (P < 0.01, Fig. 6D), indicating that TSC1 deficiency increased Th1/Th17 cytokine expression in macrophages by activating the mTOR-STAT3 pathway. On the other hand, inhibiting RORγT transcriptional activity by RORγT inverse agonist SR2211 significantly blocked LPS-induced IL-17A and IL-17F mRNA expression but failed to inhibit the enhanced IFN-γ mRNA expression in TSC1cKO macrophages (Fig. 6E). These results collectively indicate that TSC1 inhibits Th17 cytokine expression in macrophages in an STAT3-RORγT-dependent manner. To understand the intracellular signal pathway for IFN-γ production in LPS-activated TSC1cKO macrophages, we detected the Th1-related key transcription factor T-box transcription factor (T-bet) [50]. The expression of T-bet was significantly upregulated in TSC1cKO macrophages after LPS treatment, as determined by real-time PCR (Fig. 6F), suggesting the enhanced T-bet level in TSC1cKO macrophages might be involved in the enhanced IFN-γ expression after LPS stimulation.

**Figure 7.**
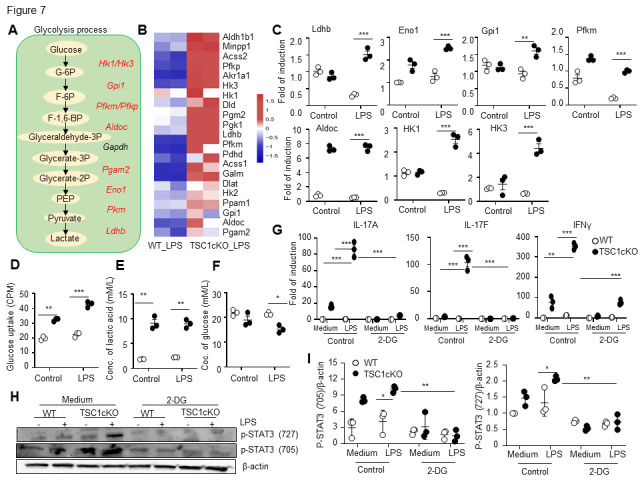
Tuberous sclerosis complex 1-deleted macrophages exhibit higher glycolytic activity than wild-type macrophages. Diagram of the up-regulated genes in the glycolytic process. Red represents increasing genes in TSC1-deleted cells (A). The expression of the changed genes after LPS treatment in the glycolysis process was performed by heatmap (B). The mRNA levels of Ldhb, Eno1, Gpi1, Pfkm, Aldoc, Hk1 and HK3 in peritoneal macrophages after Rapa pre-treatment for 90mins and then LPS treatment for 6h were detected by real-time PCR (mean ± SEM, n=3 biological replicates) (C). Glucose uptake by WT and TSC1cKO peritoneal macrophages (mean ± SEM, n=3 biological replicates) (D). Lactate levels in the culture medium of LPS-stimulated peritoneal macrophages were detected with a 1450 MicroBeta JET. F. Glucose levels in the culture medium of LPS-stimulated WT and TSC1cKO peritoneal macrophages were detected by the 1450 MicroBeta JET (mean ± SEM, n=3 biological replicates) (E). The mRNA expression of IL-17A, IL-17F, and IFN-γ in LPS and/or 2-DG-treated WT and TSC1cKO macrophages was analyzed by real-time PCR (mean ± SEM, n=3 biological replicates) (G). The levels of p-STAT3 protein were determined by western blot (H). Western blot quantification analysis by ImageJ (mean ± SEM, n=3 times experiments) (I). Data in (C) and (G) were analyzed by Students’ t-test. Data in (D-F) and (I) were analyzed by Mann-Whitney U test (*p < 0.05, **p <0.01, ***p < 0.001 compared with the control or between the indicated groups). IL, interleukin; IFN-γ, interferon gamma; TSC1, tuberous sclerosis complex 1; Aldoc, aldolase, fructose-bisphosphate C; GPI, glucose-6-phosphate isomerase; PFKM, phosphofructokinase; Eno1, enolase 1; LDH-b, lactate dehydrogenase;HK, hexokinase.

### Glycolysis contributed to Th1/Th17 cytokine expression in TSC1-deficient macrophages

It has been proposed that mTORC1-dependent metabolic reprogramming of aerobic glycolysis is especially important for Th17 cell development [51-54]. Glucose utilization depends on a chain of reactions catalyzed by multiple enzymes, eventually leading to the generation of lactate and net production of two ATP molecules as the energy source. RNA-seq analyses of TSC1cKO macrophages versus WT macrophages in the presence or absence of LPS stimulation revealed marked upregulation of genes encoding various key molecules involved in glycolysis (Fig. 7A, B). We therefore examined the expression of glycolytic enzymes hexokinase 1 (HK1), aldolase, fructose-bisphosphate C (Aldoc), glucose-6-phosphate isomerase (GPI), phosphofructokinase (PFKM), enolase 1 (Eno1), and lactate dehydrogenase (LDH-b) by real-time PCR. Consistent with the RNA-seq data, the expression levels of these glycolytic molecules were significantly higher in TSC1cKO macrophages compared with WT macrophages (Fig. 7C). In contrast to WT macrophages, TSC1cKO macrophages also showed more glucose utilization (Fig. 7D), as well as increased production of lactic acid (Fig. 7E). The concentration of glucose in cell culture supernatant of LPS-treated TSC1cKO macrophages was significantly lower than that in LPS-treated WT macrophages (Fig. 7F). These results indicate that TSC1cKO macrophages exhibit high glycolytic activity. To confirm whether the glycolysis contributes to Th1/Th17 cytokine expression in TSC1-deficient macrophages, freshly isolated peritoneal macrophages were used to investigate the hypothesis. We found that the blockade of glycolysis with 2-deoxyglucose (2-DG) decreased the IL-17A, IL-17F, and IFN-γ mRNA expression in TSC1cKO macrophages (Fig. 7G), indicating that glycolysis is involved in regulating the IL-17A, IL-17F, and IFN-γ mRNA expression in TSC1-deficient macrophages. Having established an essential role of STAT3 in controlling Th17 cytokine expression in TSC1cKO macrophages, we next wondered whether metabolic reprogramming is instrumental to the signal pathway. We found that the inhibition of glycolysis by 2-DG significantly diminished the STAT3 activation based on the enhanced levels of p-STAT3 (at amino acids Y705 and S727; Fig. 7H-I). Therefore, TSC1 deficiency made IL-17A, IL-17F, and IFN-γ expression in LPS-stimulated macrophages likely via the upregulation of the mTOR-glycolysis-STAT3-dependent pathway.

## DISCUSSION

Macrophages play a vital role in controlling the duration and magnitude of host defense against pathogenic infections and inflammatory responses [55]. In general, macrophages are classified as classical activated macrophages (M1) and alternative activated macrophages (M2), according to the cytokine milieu [56, 57]. In recent years, several studies have revealed a new unclassical inflammatory macrophage subpopulation, which has a distinct gene profile in contrast to M1 and M2, and selectively produces Th17 cytokines [17, 58, 59]. Using loss of function approach, we found TSC1 was a negative regulator of Th1/Th17-like macrophage polarization induced by LPS. Mice with myeloid-specific TSC1 deletion are hypersensitive to DSS-induced acute colitis, and the colonic macrophages of TSC1cKO mice express high levels of IL-17A, IL-17F, and IFN-γ. The present study demonstrated that mTOR hyperactivity caused by TSC1 deficiency lead to unclassical polarization of macrophages, which express high level of Th1/Th17 cytokines in response to LPS. It is well known that the loss of TSC1 or TSC2 results in hyperactivity of mTORC1. The TSC1-mTOR pathway negatively regulated classical M1 macrophage polarization in terms of TNF-α, IL-12, IL-6 and iNOS expressions. [21, 39, 60]. However, our present work showed that mTOR deletion or Rapa treatment significantly reversed the enhanced expression of IL-17A, IL-17F, and IFN-γ in TSC1cKO macrophages. Therefore, TSC1 pathway controls the classical and unclassical inflammatory polarization in LPS-activated macrophages through different signal pathways.

Genetic analysis illustrated that the cytokine interaction pathway and inflammation-associated signaling pathway were enriched in TSC1-deficient macrophages under LPS stimulation. STAT3 and its related signaling pathway were significantly upregulated in LPS-treated TSC1-deficient macrophages. It has been reported that mTOR played a crucial role in inducing Th17 differentiation by promoting STAT3 activation [61-63]. Activated STAT3 could promote transcription of RORC (encoding RORγT) to enhance the expression of pro-inflammatory effector molecules, including IL-17A and IL-17F during Th17 and N17 differentiation [64, 65]. In TSC1cKO macrophages, both STAT3 inhibitor and RORγT inhibitor significantly decreased the enhanced IL-17A and IL-17F expression in LPS-treated TSC1-deficient macrophages, indicating TSC1 deficiency induced a Th17-like cytokine profile in macrophages under LPS stimulation via the mTOR-STAT3-RORγT pathway. MTOR played an important role in activating a metabolic regulatory network by controlling the crucial transcription factors in glycolysis [3, 7]. Consistent with the published studies, we demonstrated that the glycolysis gene profile was characteristically enriched in the LPS-stimulated TSC1-deficient macrophages. TSC1-deficient macrophages displayed higher glucose consumption and produced more lactic acid. The glycolytic enzyme PKM2 and hypoxia-inducible factor 1 alpha promote Th17 cell differentiation by fine-tuning STAT3 activation [51, 54]. 2-DG treatment inhibited STAT3 activation and decreased the expression of IL-17A, IL-17F, and IFN-γ in LPS-treated TSC1cKO macrophages. These studies collectively indicated that TSC1 regulated IL-17A, IL-17F, and IFN-γ expression in LPS-treated macrophages via the glycolysis-STAT3-RORγT pathway.

In addition, IFN-γ is usually produced by NK, Th1 cells, and other immune cells. Monocytes/macrophages could express IFN-γ after IL-12/IL-18, LPS/ATP, and IL-23 stimulation, respectively [17, 66]. LPS stimulation alone could not efficiently induce IFN-γ expression in macrophages in early stage. However, in the present study, we found that LPS stimulation induced high level of IFN-γ expression in TSC1-deficient macrophages. Molecular mechanism studies showed that the expression of IFN-γ in LPS-stimulated TSC1-deficient macrophages was mediated by T-bet pathway rather than RORγT pathway. Thus, TSC1 expression is essential for WT macrophages to turn off T-bet and to block IFN-γ expression during LPS stimulation.

In conclusion, we herein demonstrated that TSC1-mTOR signaling pathway negatively regulated Th1/Th17-type cytokine expression in LPS-stimulated macrophages and exacerbated pathogenesis of colitis in mice via reprogramming metabolism and STAT3-RORγT/T-bet pathway. The identification of the master role of TSC1 in silencing Th1/Th17-type cytokine expression in LPS-stimulated macrophages provided a new molecular mechanism for the unclassical polarization of macrophages and might offer novel approaches to prevent macrophage hyperactivation-related diseases.
